# Southernmost records of *Escarpia spicata* and *Lamellibrachia barhami* (Annelida: Siboglinidae) confirmed with DNA obtained from dried tubes collected from undiscovered reducing environments in northern Chile

**DOI:** 10.1371/journal.pone.0204959

**Published:** 2018-10-09

**Authors:** Genki Kobayashi, Juan Francisco Araya

**Affiliations:** 1 Atmosphere and Ocean Research Institute, The University of Tokyo, 5-1-5 Kashiwanoha, Kashiwa, Chiba, Japan; 2 Centro de Investigaciones Costeras de la Universidad de Atacama (CIC-UDA), Universidad de Atacama, Copayapu 485, Copiapó, Región de Atacama, Chile; 3 Programa de Doctorado en Sistemática y Biodiversidad, Universidad de Concepción, Concepción, Chile; Nanjing Agricultural University, CHINA

## Abstract

Deep-sea fishing bycatch enables collection of samples of rare species that are not easily accessible, for research purposes. However, these specimens are often degraded, losing diagnostic morphological characteristics. Several tubes of vestimentiferans, conspicuous annelids endemic to chemosynthetic environments, were obtained from a single batch of deep-sea fishing bycatch at depths of around 1,500 m off Huasco, northern Chile, as part of an ongoing study examining bycatch species. DNA sequences of the mitochondrial cytochrome *c* oxidase subunit I (COI) gene and an intron region within the hemoglobin subunit B2 (hbB2i) were successfully determined using vestimentiferans’ dried-up tubes and their degraded inner tissue. Molecular phylogenetic analyses based on DNA sequence identified the samples as *Escarpia spicata* Jones, 1985, and *Lamellibrachia barhami* Webb, 1969. These are the southernmost records, vastly extending the geographical ranges of both species from Santa Catalina Island, California to northern Chile for *E*. *spicata* (over 8,000 km), and from Vancouver Island Margin to northern Chile for *L*. *barhami* (over 10,000 km). We also determined a 16S rRNA sequence of symbiotic bacteria of *L*. *barhami*. The sequence of the bacteria is the same as that of *E*. *laminata*, *Lamellibrachia* sp. 1, and *Lamellibrachia* sp.2 known from the Gulf of Mexico. The present study provides sound evidence forthe presence of reducing environments along the continental margin of northern Chile.

## Introduction

Deep-sea fishing bycatch provides a glimpse into the species co-occurring with commercial fishes and often comprises a way of recording rare species that are not easily accessible for research. However, bycatch is seldom reported, kept, or landed due to a lack of commercial interest, administrative restrictions, and for other socio-economic reasons [1]. In addition to the inconsistent and fragmented nature of these records, examining such organisms is often hampered by their degradation before they reach researchers. Most often, the only available remains of deep-sea fishing bycatch are carapaces or shells, or, in the case of vestimentiferan tubeworms, their hardened chitin-protein tubes. Therefore, identification of vestimentiferans to species level is impeded by the absence of diagnostic soft parts.

Vestimentiferans are interesting members of the annelid family Siboglinidae as they lack a mouth and digestive organs, depending on endosymbiotic chemoautotrophic bacteria for nutrition in the adult phase [2]. To date, 20 vestimentiferan species within 10 genera have been recorded from hydrothermal vent fields [3, 4], cold-seep areas [5, 6], and organic falls [7–9]. In the Pacific Ocean, vestimentiferans are particularly diverse, with four genera identified in cold-seep areas: *Alaysia* Southward,1991; *Escarpia* Jones, 1985; *Lamellibrachia* Webb, 1969; and *Paraescarpia* Southward, Schulze & Tunnicliffe, 2002 [3, 10–12]. While the monotypic genus *Alaysia* is known from a hydrothermal vent field,several undescribed species of the genus have been collected from cold-seep areas around Japan [13]. *Escarpia* includes three described and a few undescribed species [14, 15]; *Lamellibrachia*, the most diverse group among vestimentiferans, consists of eight named and several species so far undescribed [16–18], while the genus *Paraescarpia* is monospecific.

In the cold-seep areas of the northeastern Pacific, two vestimentiferan species have been reported: *Escarpia spicata* Jones, 1985, known from off Santa Catalina Island, California to Middle American Trench (reviewed by Karaseva et al. [19]); and *Lamellibrachia barhami* Webb, 1969, known from British Columbia to Costa Rica (reviewed by Karaseva et al. [19]). However, to date, no vestimentiferan species have been identified to the species level in the southeastern Pacific. A record of “pogonophoran,” which may represent a vestimentiferan species, was reported from a seep site in the Peruvian margin, off Paita ~5°S [20], while an unidentified vestimentiferan species was recorded from the Concepción Methane Seep Area (CMSA) off Concepción ~36°S [21]. This undescribed species is suggested to be most closely related to *Lamellibrachia luymesi* van der Land & Nørrevang, 1975 [22], described from the Gulf of Mexico, based on the partial sequence of the mitochondrial cytochrome *c* oxidase subunit I (COI) [21]. Furthermore, empty tubes of vestimentiferans have also been collected off Chile nearby the Taitao Peninsula, ~46°S [23], and off El Quisco, ~33°S [24].

Some vestimentiferan tubes lacking diagnostic soft parts were also collected as bycatch of deep-sea fishing off Huasco, northern Chile, in September 2017. As part of an ongoing project investigating the bycatch of deep-sea fishing in northern Chile [25–31],the present study reports two species of vestimentiferans, identified to the species level through molecular phylogenetic analyses based on DNA sequences determined using dried-up tubes and tissue.

## Materials and methods

### Sampling

Ten anterior parts and some fragments of vestimentiferan tubes lacking posterior parts were collected as bycatch of longline fishing by the fisheries vessel (FV) Rocio III during fishing of *Dissostichus eleginoides* Smitt, 1898 (Patagonian toothfish or Chilean sea bass) fishing, at a depth of about 1,500 m off Huasco (28°S, 71°W; accurate coordinates are not available), Región de Atacama, northern Chile, in September 2017. As this material was serendipitously collected in the fish bycatch (discarded material), no permit was necessary for the current research. Siboglinids are not endangered nor protected by local law. The substrata of tubes were not collected. Four tube samples (two anterior parts and two fragments) containing degraded tissues (may be trophosome of the worms) were used for morphological and molecular analyses. The samples were stored at room temperature in Chile (around 18°C) from September 2017 to March 2018, after which they were used for DNA extraction. Following DNA extraction, four specimens were preserved at −20°C.

### Polymerase chain reaction (PCR) and sequencing

Total DNA was extracted both from the tissue left inside each of four tubes (probably consisting of trophosomes) and from the tubes themselves, using a DNeasy Blood and Tissue Kit (QIAGEN, Hilden, Germany), following a normal protocol of manufacturer’s recommendations. Tube pieces were carefully cut from parts of the tube where obvious tissue were absent, but see the “DNA extraction from dried-up vestimentiferan tubes” section in Discussion. Fragments of the mitochondrial COI gene (658 bp) were amplified by PCR using a primer set LCO1490 (5′-GGTCAACAAATCATAAAGATATTGG-3′) and HCO2198 (5′-TAAACTTCAGGGTGACCAAAAAATCA-3′) [32]. Fragments of an intron region within the hemoglobin subunit B2 (hbB2i; ~660 bp) were amplified with the following primer sets: hbB2i_F (5′-TCCATCGCCCCAGGCTGTCTTC-3′); and hbB2i_R (5′-GCCTTGAATTCGTTGCTGTT-3′) [33]. A mitochondrial gene (16S rRNA; 1409 bp) of symbiotic bacteria was amplified with primer set 27F (5′-AGAGTTTGATCMTGGCTCAG-3′) and 1492R (5′-TACGGYTACCTTGTTACGACTT-3′) [34].

The PCR mixtures for vestimentiferans contained 16 μl DDW, 0.13 μl TaKaRa Ex Taq Hot Start Version (TaKaRa Bio Inc., Kusatsu, Japan), 2.5 μl 10× Ex Taq Buffer, 2.0 μl dNTP mixture (2.5 μM each), 0.3 μl forward and reverse primers (20 μM each), and 4.0 μl template DNA. For bacteria, the PCR mixtures contained 7.3 μl DDW, 0.1 μl TaKaRa Ex Taq Hot Start Version, 1.3 μl 10× Ex Taq Buffer, 1.0 μl dNTP mixture (2.5 μM each), 0.65 μl forward and reverse primers (10 μM each), and 2.0 μl template DNA. PCR amplifications were performed as follows: initial denaturation at 94°C for 120 s; followed by 35 cycles consisting of denaturation at 94°C for 30 s, annealing at 42°C (COI) or 53°C (hbB2i) for 40 s, extension at 72°C for 20 s; and a final extension at 72°C for 300 s. Exceptions included annealing at 52°C for 20 s and 105 s of extension for bacterial 16S rRNA. Obtained PCR products were purified with ExoSAP-IT (Thermo Fisher Scientific, Waltham, MA) and then sequenced using the same primer sets asforPCR. Sequencing reactions were prepared using a BigDye Terminator Cycle Sequence Kit v3.1 (Applied Biosystems [ABI], Foster City, CA). Nucleotide sequences were determined using an ABI 3130xl automated DNA sequencer after being purified with a BigDye XTerminator Purification Kit (ABI).

### Phylogenetic analysis

A total of 71 COI sequences of vestimentiferans and two sequences of other siboglinid species were used for phylogenetic analysis. Accession numbers obtained from GenBank are shown after the taxonomic names in the resultant tree. There were no indels resulting in an unambiguous alignment for the COI. Phylogenetic trees were reconstructed using Bayesian inference and maximum likelihood (ML) methods, based on the COI dataset. Bayesian analysis was performed using MrBayes v3.1.2. [35], with the setting “branch lengths unlinked.” Partitioning scheme and best-fit substitution models were estimated using PartitionFinder v2.1.1. [36] with “model selection” set to “AICc,” “branchlengths” set to “unlinked,” and using the “-raxml” option [37]: TRN + Γ+ I for the first + second codon positions of COI; GTR + Γ + I for the third codon position of COI. Since the TRN model was not implemented in MrBayes,it was replaced by the GTR model. Two parallel runs were made for 5,000,000 generations (with a sampling frequency of 1,000), using the default value of four Markov chains. The initial 25% of samples were discarded, and the subsequent 75% were used to confirm that the four chains reached stationary distributions, referring to the average standard deviation of split frequencies [35]. The ML analysis was performed using RAxML v7.2.6 [37]. The rapid bootstrap analysis was used to identify the best-scoring ML tree in a single program run, and to identify 500 bootstrap replicates under the GTR + Γ + I substitution model for all partitions.

DNA sequences of hbB2i of 42 *Escarpia* species and of five *Seepiophila jonesi* (outgroup) were aligned using MAFFT v7.294b with the default option [38]. Only three bp of a deletion was found in *S*. *jonesi*, resulting in non-ambiguous alignment. A phylogenetic analysis was conducted to identify the *Escarpia* specimens to the species level, based on the hbB2i sequences. Bayesian inference and ML methods were employed with the same options as the analysis for the COI genes. The GTR + G model was estimated as the best-fit substitution model with PartitionFinder v2.1.1. for the Bayesian analysis. All trees were edited using FigTree v1.4.3 (http://tree.bio.ed.ac.uk/software/figtree/).

## Results

### Tube morphology and associated species

**Fig 1:**
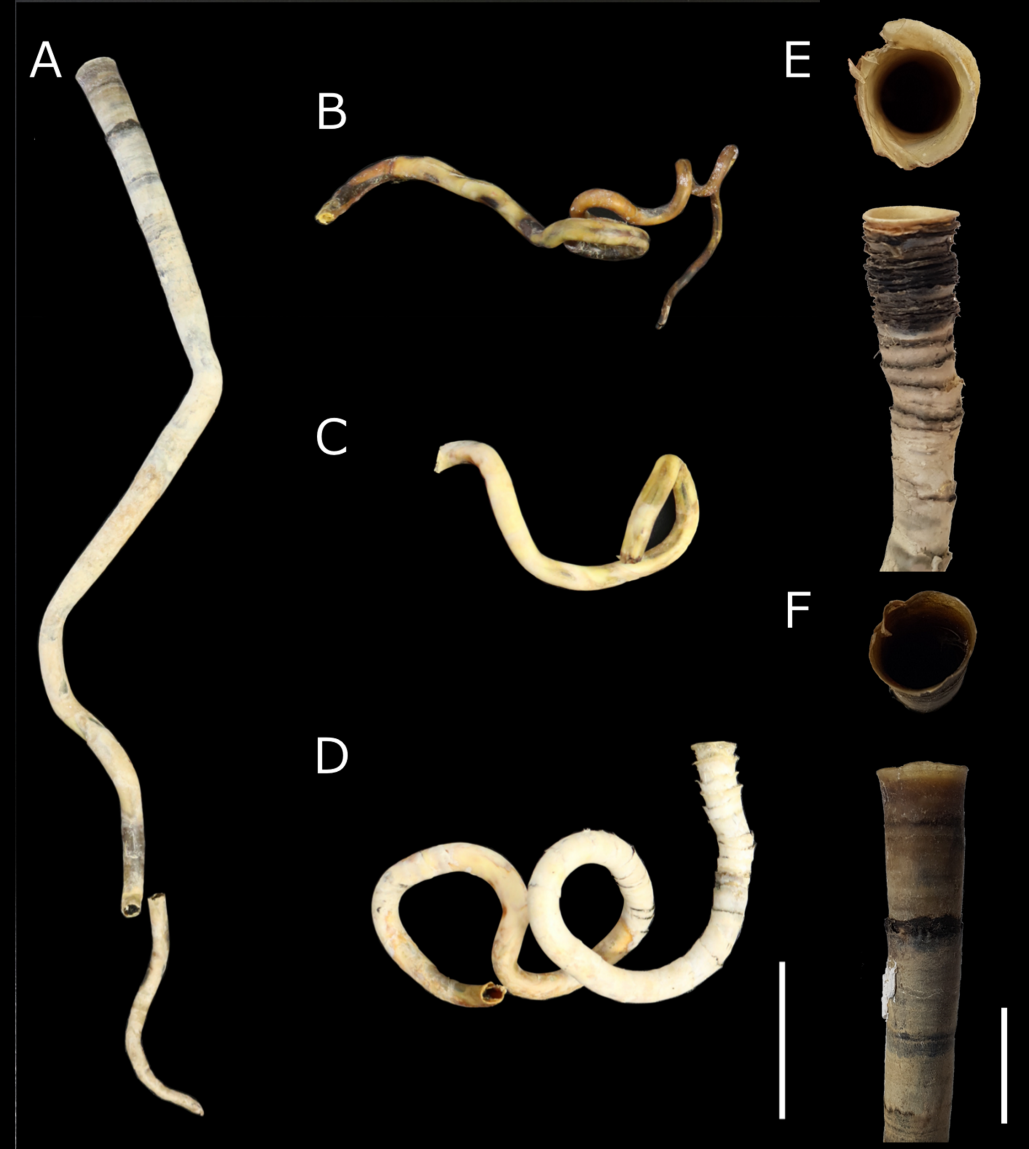
Vestimentiferan tubes. *Escarpia spicata* (A, GK608); *Lamellibrachia barhami* (B, GK607; C, GK605; D, GK621); other vestimentiferan tubes which were not used for molecular analyses (E, F). Scale bar = 5 cm for A–D, 2 cm for E and F.

The tubes of GK608 and GK621 included anterior regions but lacked posterior ones, whereas those of GK605 and GK607 lacked both anterior and posterior regions. For GK621, the outer width of the top funnel opening was 14.9 mm,whereas its base was 11.7 mm (Fig 1). Unlike the other tubes, GK608 did not form conspicuous funnels, presenting a smooth surface (Fig 1) with its top opening measured at 14.0 mm. The only organisms attached to the tubes were unidentified species of limpets.

### PCR amplification of the DNA extracted from vestimentiferan tissue and tubes

DNA sequences were successfully obtained from the dried-up three vestimentiferan tissue (GK605, GK607, and GK608) and two tubes (GK605 and GK621). The partial sequences of the mitochondrial COI (658 bp) of GK605 and GK621 were identical, making it impossible to determine whether the specimens were derived from different individuals. Although PCR was successful for the other tissue and tube samples, the sequences were not determined by direct sequencing.

### Phylogenetic analyses of vestimentiferans

**Fig 2:**
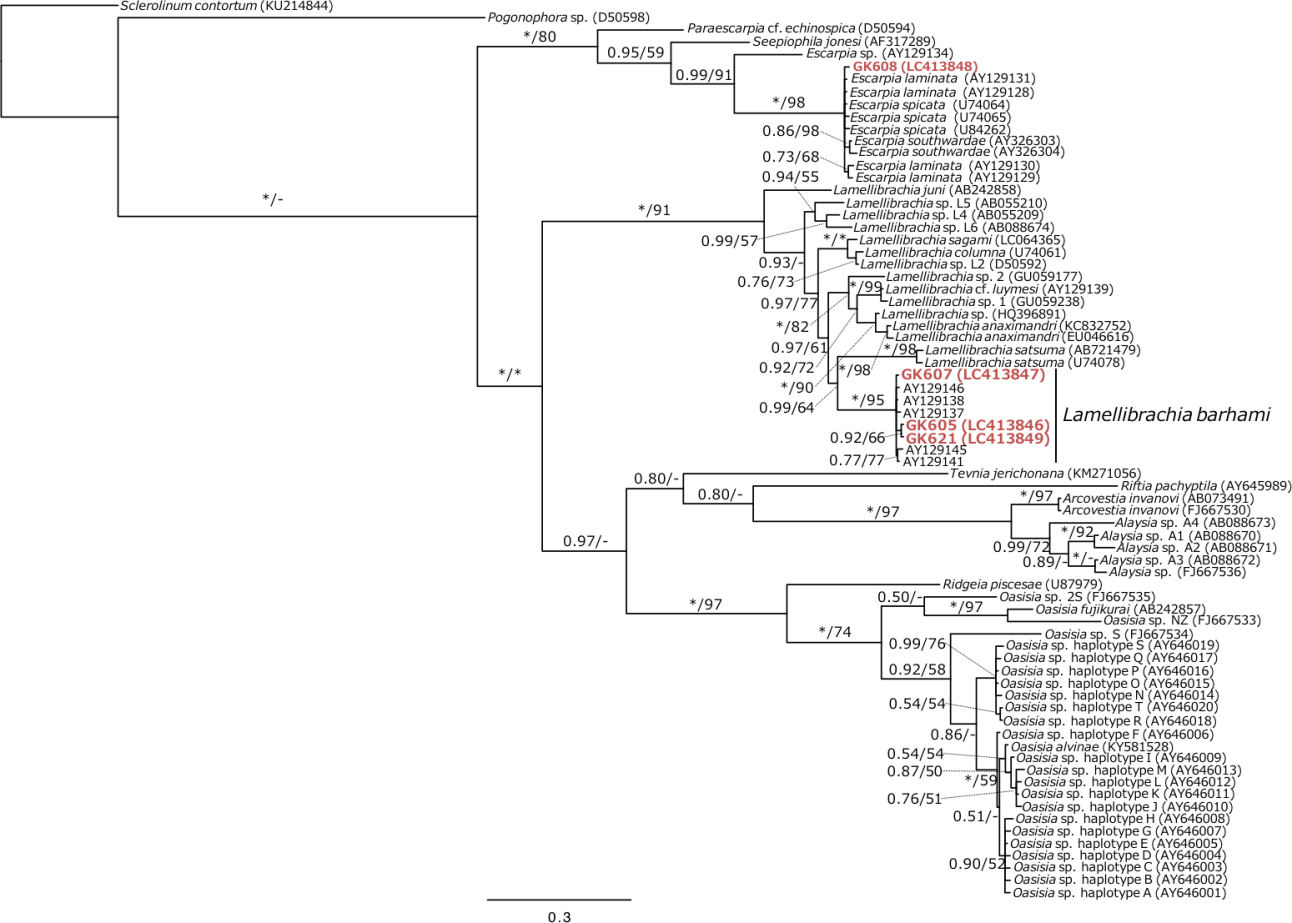
Bayesian phylogeny of vestimentiferans based on the COI gene sequences (up to 1270 bp). The numbers above the branches indicate the posterior probability (PP), followed by the percentage of maximum likelihood bootstrap probabilities (BS) above 50%. Asterisks indicate values of 1.00 (PP) or 100% (BS) and hyphens do value below 50% (BS).

**Fig 3:**
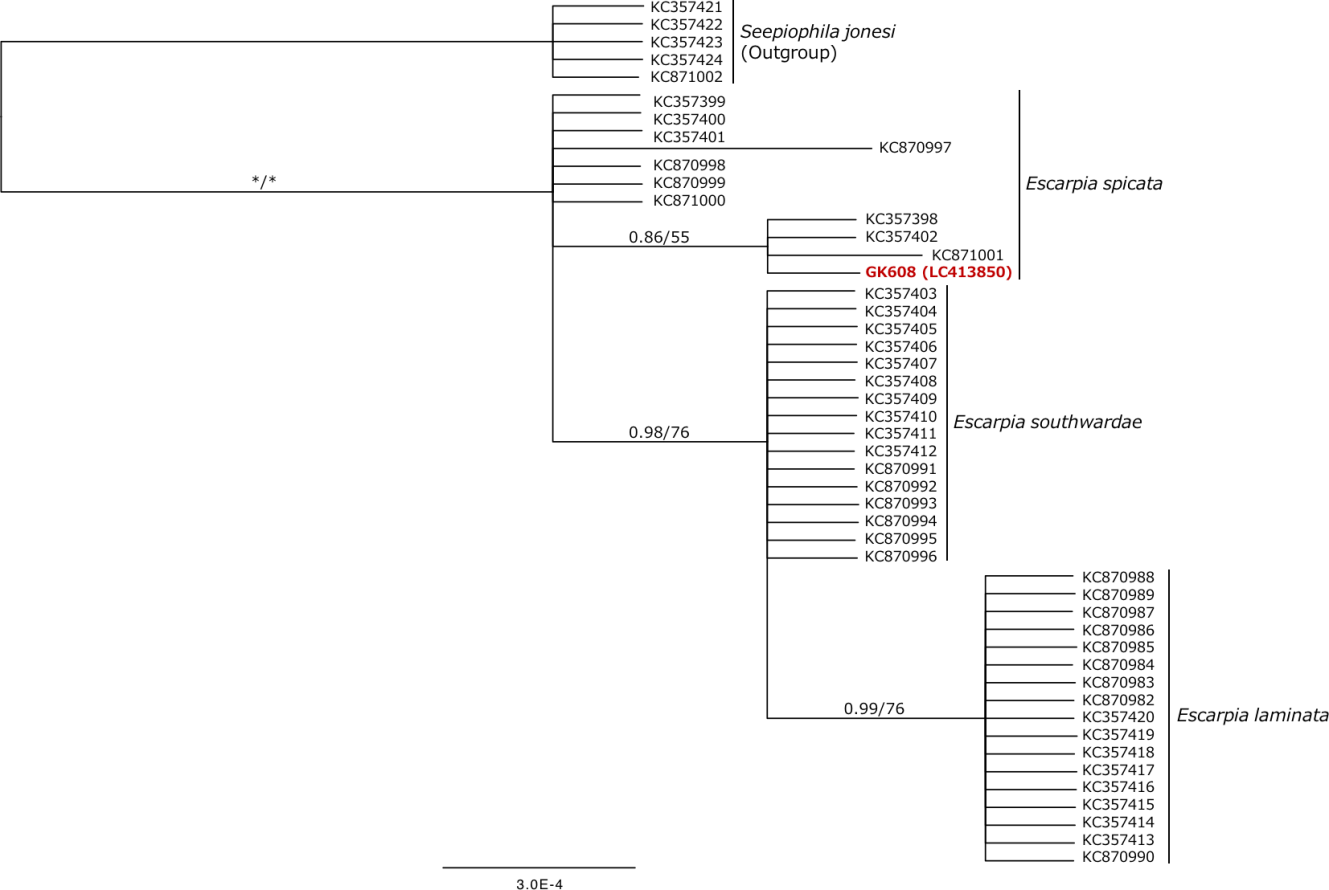
Bayesian phylogeny of *Escarpia* species based on the hbB2 gene sequences (up to 660 bp). *Seepiophila jonesi* was included as an outgroup. The numbers above the branches indicate the posterior probability (PP), followed by the percentage of maximum likelihood bootstrap probabilities (BS) above 50%. Asterisks indicate values of 1.00 (PP) or 100% (BS).

Since Bayesian and ML analyses of the COI dataset generated similar tree topologies and support values, only the Bayesian tree is shown with posterior probabilities (PP) and ML bootstrap values (BS) (Fig 2). As shown in Fig 2, all sequences of vestimentiferans collected from off the coast of northern Chile were included in highly supported clusters. Three of these vestimentiferans (GK605, GK607, and GK621) were clustered with *Lamellibrachia barhami* (PP = 1.00, BS = 95%), while the other vestimentiferan (GK608) was included in a cluster comprising *Escarpia laminata* Jones, 1985; *Escarpia southwardae* Andersen, Hourdez, Jolivet, Lallier and Sibuet, 2004; and *Escarpia spicata* (PP = 1.00, BS = 98%). The phylogenetic relationships of the *Escarpia* species are not clear from the COI tree, as *E*. *laminata* and *E*. *spicata* were not monophyletic. The Bayesian tree based on the hbB2i sequences of the *Escarpia* species showed that a single cluster was recovered for *E*. *laminata* (PP = 0.99, BS = 76%) and a sub-cluster, which includes four sequences, was recognized for *E*. *spicata* with weak support values (PP = 0.86, BS = 55%), whereas clusters were not recovered for *E*. *southwardae* nor the rest of *E*. *spicata* (Fig 3). GK608 was included in the *E*. *spicata* clade.

### DNA sequence of symbiotic bacteria

Direct sequencing allowed a 16S rRNA sequence of symbiotic bacteria to be obtained from the tissue of vestimentiferan GK607. No variable sites were found between the sequence of symbiotic bacteria of GK607 and that of *Escarpia laminata* (Accession No. HE983329, 1335 bp), *Lamellibrachia* sp. 1 (HE983327; 1471 bp), and *Lamellibrachia* sp. 2 (HE983328, 1372 bp; HE983337, 1279 bp), all of which were collected from the Gulf of Mexico at depths of 2,335–2,604 m [39]. The 16S rRNA sequence of symbiotic bacteria obtained fromGK607 is almost same as the sequence of symbiotic bacteria obtained from *Lamellibrachia barhami* (AY129103, 1361 bp) collected from the Vancouver Island Margin at a depth of 1,300 m, with only a single nucleotide substitution [40].

## Discussion

### Tube morphology

Tube morphology was insufficient to identify vestimentiferan specimens to the species level. All tubes presented a hard, wood-like texture, similar to those of vestimentiferans inhabiting cold-seep areas, including species of the genera *Escarpia*, *Lamellibrachia*, *Paraescarpia*, and *Seepiophila*. The samples GK605 and G607 lacked both anterior and posterior parts, making species identification from tube morphology impossible. A coiled tube with funnels (GK621) resembled that of *Lamellibrachia barhami*, shown in fig 25 by Webb [41]. Such entangled tubes are unknown in any other vestimentiferans, especially in terms of anterior region of tubes. Although a smooth, straight tube, lacking evident funnels (GK608) resembles those of *Escarpia* species [3, 15]; some *Lamellibrachia* species also lack conspicuous funnels in the anterior region [10, 18]. Moreover, vestimentiferans show considerable plasticity in terms of tube morphology [6, 42, 43], making identification to the species level from tube morphology difficult.

### DNA extraction from dried-up vestimentiferan tubes

DNA sequences were successfully obtained from vestimentiferans dried-up tissue and tubes. Although a previous study extracted DNA from the degraded tissue of *Lamellibrachia* species [44], to our knowledge the present study is the first time that DNA is successfully amplified from vestimentiferan tubes, which are constituted by chitin and protein secreted by vestimentiferans [45, 46]. Discarded tissues that are included in secretions, such as mucus, can be a source of DNA [47]. By experimentally immersing *Riftia* tubes into a hydrothermal vent field for 180 days, *Riftia* tubes were estimated to degrade within 2.5 years of the death of the organism [48]. DNA extraction from tubes may, therefore, allow identification of the vestimentiferan species, by using vacant tubes without the soft parts, which usually prevents identification to the species level. Although the tubes which were used in the present study were carefully cut to exclude dried tissue from DNA extraction, in future studies DNA extraction using degraded tubes that is completely free from remains of vestimentiferan tissue would be needed, to eliminate false positives for DNA present in the tubes.

### Identification based on molecular phylogeny of vestimentiferans from Chile

The COI phylogenetic tree shows that three vestimentiferan sequences (GK605, GK607, and GK621) were clustered with *Lamellibrachia barhami* with high support values (Fig 2). Although one vestimentiferan sequence (GK608) was clustered with three *Escarpia* species, the COI analysis did not allow for identification to the species level (Fig 2). The specimen was clustered with *Escarpia spicata* in the hhBb2i phylogenetic tree (Fig 3). The hhBb2i sequence is known as a useful indicator to identify the three *Escarpia* species, which are not discriminated by COI sequences [33]. Thus, the present results allow the identification of the vestimentiferan tubes collected from off Chile confidently to be *L*. *barhami* and *E*. *spicata*.

### Implications for vestimentiferan biogeography and phylogeography

The present study generated new records of two vestimentiferan species from the Chilean waters, including the first record of the genus *Escarpia*. *Lamellibrachia barhami* was previously identified along the continental margin of the northeastern Pacific from the Vancouver Island Margin to Costa Rica, at depths of 1,000–2,400 m (reviewed by Karaseva et al. [19]; they regarded the “Vigo worm” collected from off Spain as *L*. *barhami*, although the 28S rRNA sequence of the Vigo worm considerably differs from that of *L*. *barhami* [44], thus we did not include the record from off Spain here), at both hydrothermal vent fields and cold-seep areas. *Escarpia spicata* was previously identified in chemosynthetic environments from off Santa Catalina Island, California, to the Middle American Trench at depths of 1,240–2,756 m (reviewed by Karaseva et al. [19]). The present record of both species considerably extends their southernmost limit: the geographic range of *L*. *barhami* is over 10,000 km for straight-line distance and that of *E*. *spicata* is over 8,000 km. Vestimentiferans inhabiting hydrothermal vent fields (i.e., *Riftia pachyptila* Jones, 1981; and *Tevnia jerichonana* Jones, 1985) present a wide geographical distribution across the eastern Pacific [19]; the present study provides the first record of cold-seep vestimentiferans with a broad distribution across the eastern Pacific.

Despite an 8,000 km distance, *L*. *barhami* from Monterey Canyon (e.g., AY129137, AY129138) and from Chile (GK607, LC413847) are identical in terms of shared sites (633 bp) of the COI gene, indicating a close intra-specific relationship, similar to that of other eastern Pacific vestimentiferans (e.g., *R*. *pachyptila* and *T*. *jerichonana)* for which shared COI haplotypes were reported between specimens from the northeastern and southeastern Pacific, although different haplotypes dominate at north and southern localities [49, 50]. This little genetic divergence in the COI gene may be attributed to the slow evolutionary rate in the gene [40] or to a recent radiation of vestimentiferan species. In general, deep-sea benthic invertebrates show a wide geographical distribution with little genetic divergence [50–54], and the present study provides another example of such a pattern. Further analyses including more specimens are needed to further discuss the phylogeography of *L*. *barhami*. Unfortunately, there are still no appropriate DNA markers available for intra-specific phylogeography of *E*. *spicata*.

Vestimentiferan species play an important role in structuring the benthic community by providing microhabitats for other organisms [55, 56]. The chitinous tubes of vestimentiferans increase the spatial heterogeneity in soft bottoms and are used as substrata for colonization of various epibenthos, in terms of their taxon and body size [57–64]. Although only unidentified limpets were found in the surfaces of examined tube specimens, hidden communities of these vestimentiferans would harbor epibenthos and extend their southern limits.

### Symbiotic bacteria of *Lamellibrachia barhami* from off Chile

The 16S rRNA sequence of symbiotic bacteria was determined through direct sequencing of the total DNA extracted from the degraded tissue of *L*. *barhami* (GK607). Although vestimentiferans host multiple symbiont lineages [65], Gammaproteobacteria are dominantly present in the trophosome, thus their sequences may be determined by direct sequencing. The present sequence was identical to Gammaproteobacteria-affiliated 16S rRNA sequences obtained from *E*. *laminata*, *L*. sp. 1, and *L*. sp. 2 from the Gulf of Mexico [66]. A similarity in the sequences of symbiotic bacteria of GK607 and those of vestimentiferans inhabiting the Gulf of Mexico support previous reports that close relationships have been shown for symbiotic bacteria of vestimentiferans separated by great distances [40, 66].

## Conclusions

The present study represents an additional case study that the bycatch of deep-sea commercial fishing provides valuable information about rare species (see Introduction).

We successfully extracted total DNA from dried-up tissue and tubes of vestimentiferans, and showed that dried tubes, in addition to degraded tissue [44], are usable to obtain DNA. The Molecular phylogenetic analysis based on the COI gene successfully identified *Lamellibrachia* specimens, which are difficult to identify from the morphological characters of tubes. In addition to the COI gene, the hbB2i sequences were useful to identify the *Escarpia* species, as was reported by Cowart et al. [33]. Although the duration of DNA in the vestimentiferan tubes remains unknown, extracting DNA from the tubes is thus useful to identify tube-building species.

Our records of *E*. *spicata* and *L*. *barhami* from Chile considerably extend the previously-known geographic distribution of these two species; *E*. *spicata* was previously known to exist north of Mexico, whereas *L*. *barhami* was known to exist north of Costa Rica. A patchy distribution of reducing environments may account for the sparse records of vestimentiferans in the southeastern Pacific. A broad geographic species distribution is, however, not uncommon among deep-sea organisms, sometimes through a whole stretch of a submarine ridge or a continental margin [49, 50, 52].

The presence of these vestimentiferans provides a sound evidence for the occurrence of reducing environments along the continental margin in the northern Chile. Heterogeneous environments may partly explain the high biodiversity existing in the fishing grounds of *Dissostichus eleginoides*, whose habitat is related to such reducing environments [67].

## References

[1] Arana PM, Wehrtmann IS, Orellana JC, Nielsen-Muñoz V, Villalobos-Rojas F. By-catch associated with fisheries of *Heterocarpus vicarius* (Costa Rica) and *Heterocarpus reedi* (Chile) (Decapoda: Pandalidae): a six-year study (2004–2009). J Crustacean Biol. 2013; 33:198–209. 10.1163/1937240X-00002123

[2] Bright M, Lallier FH. The biology of vestimentiferan tubeworms. Oceanogr Mar Biol. 2010; 48: 213–266.

[3] Jones ML. On the Vestimentifera, new phylum: six new species, and other taxa, from hydrothermal vents and elsewhere. Bull Biol Soc Wash. 1985; 6: 117–158

[4] Juniper SK, Tunnicliffe V, Southward EC. Hydrothermal vents in turbidite sediments on a Northeast Pacific spreading centre: organisms and substratum at an ocean drilling site. Can J Zool. 1992; 70: 1792–1809. 10.1139/z92-247

[5] Baco AR, Rowden AA, Levin LA, Smith CR, Bowden DA. Initial characterization of cold seep faunal communities on the New Zealand Hikurangi margin. Mar Geol. 2010; 272: 251–259. 10.1016/j.margeo.2009.06.015

[6] Southward EC, Andersen AC, Hourdez S. *Lamellibrachia anaximandri* n. sp., a new vestimentiferan tubeworm (Annelida) from the Mediterranean, with notes on frenulate tubeworms from the same habitat. Zoosystema. 2011; 33: 245–279. 10.5252/z2011n3a1

[7] Dando PR, Southward AJ, Southward EC, Dixon DR, Crawford A, Crawford M. Shipwrecked tube worms. Nature 1992; 356: 667 10.1038/356667a0

[8] Hughes DJ, Crawford M. A new record of the vestimentiferan *Lamellibrachia* sp. (Polychaeta: Siboglinidae) from a deep shipwreck in the eastern Mediterranean. Mar Biodivers Rec. 2008; 1: e21 10.1017/S1755267206001989

[9] Gambi MC, Schulze A, Amato E. Record of *Lamellibrachia* sp. (Annelida: Siboglinidae: Vestimentifera) from a deep shipwreck in the western Mediterranean Sea (Italy). Mar Biodivers Rec. 2011; 4: e24 10.1017/S1755267211000261

[10] Southward EC. Three new species of Pogonophora, including two vestimentiferans, from hydrothermal sites in the Lau Back-arc Basin (Southwest Pacific Ocean). J Nat Hist. 1991; 25: 859–881. 10.1080/00222939100770571

[11] Webb M. Studies on *Lamellibrachia barhami* (Pogonophora) II. The reproductive organs. Zool Jb Anat Bd. 1997; 97: 455–481.

[12] Southward EC, Schulze A, Tunnicliffe V. Vestimentiferans (Pogonophora) in the Pacific and Indian Oceans: a new genus from Lihir Island (Papua New Guinea) and the Java Trench, with the first report of *Arcovestia ivanovi* from the North Fiji Basin. J Nat Hist. 2002; 36:1179–1197. 10.1080/00222930110040402

[13] Kojima S, Ohta S, Yamamoto T, Yamaguchi T, Miura T, Fujiwara Y, Fujikura K, Hashimoto J. Molecular taxonomy of vestimentiferans of the western Pacific, and their phylogenetic relationship to species of the eastern Pacific III. *Alaysia*-like vestimentiferans and relationships among families. Mar Biol. 2003; 142: 625–635. 10.1007/s00227-002-0954-y

[14] Kojima S, Ohta S, Yamamoto T, Miura T, Fujiwara Y, Fujikura K, Hashimoto J. Molecular taxonomy of vestimentiferans of the western Pacific and their phylogenetic relationship to species of eastern Pacific. II. Families Escarpiidae and Arcovestiidae. Mar Biol. 2002; 141: 57–64. 10.1007/s00227-002-0818-5

[15] Andersen AC, Hourdez S, Marie B, Jollivet D, Lallier FH, Sibuet M. *Escarpia southwardae* sp. nov., a new species of vestimentiferan tubeworm (Annelida, Siboglinidae) from West African cold seeps. Can J Zool. 2004; 82:980–999.

[16] Kojima S, Ohta S, Yamamoto T, Miura T, Fujiwara Y, Hashimoto J. Molecular taxonomy of vestimentiferans of the western Pacific and their phylogenetic relationship to species of eastern Pacific. I. Family Lamellibrachiidae. Mar Biol. 2001; 139: 211–219.

[17] Cowart DA, Halanych KM, Schaeffer SW, FisherCR. Depth-dependent gene flow in Gulf of Mexico cold seep *Lamellibrachia* tubeworms (Annelida, Siboglinidae). Hydrobiologia. 2014; 736:139–154. 10.1007/s10750-014-1900-y

[18] Kobayashi G, Miura T, Kojima S. *Lamellibrachia sagami* sp. nov., a new vestimentiferan tubeworm (Annelida: Siboglinidae) from Sagami Bay and several sites in the northwestern Pacific Ocean. Zootaxa. 2015; 4018: 97–108. doi: 10.11646/zootaxa.4018.1.5 26624030 10.11646/zootaxa.4018.1.5

[19] Karaseva NP, Rimskaya-Korsakova NN, Galkin SV, Malakhov VV. Taxonomy, geographical and bathymetric distribution of vestimentiferan tubeworms (Annelida, Siboglinidae). Biol Bull. 2016; 43: 937–969. 10.1134/S1062359016090132

[20] Olu K, Duperret A, Sibuet M, Foucher J-P, Fiala-Medioni A. Structure and distribution of cold seep communities along the Peruvian active margin: relationship to geological and fluid patterns. Mar Ecol Prog Ser. 1996; 132: 109–125.

[21] Sellanes J, Quiroga E, Neira C. Megafauna community structure and trophic relationships at the recently discovered Concepción Methane Seep Area, Chile, ∼36°S. ICES J Mar Sci. 2008; 65: 1102–1111. 10.1093/icesjms/fsn099

[22] van der Land J, Nørrevang A. The systematic position of *Lamellibrachia* (Annelida, Vestimentifera). Z Zool Syst Evol.1975; 1: 86–101.

[23] Zapata-Hernández G, Sellanes J, Thurber AR, Levin LA. Trophic structure of the bathyal benthos at an area with evidence of methane seep activity off southern Chile (~45°S). J Mar Biol Assoc U.K. 2014; 94: 659–669. 10.1017/S0025315413001914

[24] Krylova EM, Sellanes J, Valdés F, D’Elía G. *Austrogena*: a new genus of chemosymbiotic bivalves (Bivalvia; Vesicomyidae; Pliocardiinae) from the oxygen minimum zone off central Chile described through morphological and molecular analyses. Syst Biodivers. 2014; 12: 225–246. 10.1080/14772000.2014.900133

[25] Reiswig H, Araya JF. A review of the Hexactinellida (Porifera) of Chile, with the first record of *Caulophacus* Schulze, 1885 (Lyssacinosida: Rossellidae) from the Southeastern Pacific Ocean. Zootaxa. 2014; 3889: 414–428. doi: 10.11646/zootaxa.3889.3.4 25544276 10.11646/zootaxa.3889.3.4

[26] Araya JF. New records of deep-sea spiders (Chelicerata: Pycnogonida) in the southeastern Pacific. Mar Biodivers. 2016; 46: 725–729. 10.1007/s12526-015-0416-7

[27] Araya JF, Araya ME, Mack M & Aliaga JA. On the presence of *Distichoptilum gracile* Verrill, 1882 (Octocorallia: Pennatulacea) in the southeastern Pacific. Mar Biodivers. 2018; 48(3): 1637–1641. 10.1007/s12526-016-0616-9

[28] Araya JF, Aliaga JA & Araya ME. First record of *Lillipathes ritamariae* Opresko and Breedy, 2010 (Cnidaria: Antipatharia) in the southeastern Pacific Ocean. Mar Biodivers. 2018; 48(3): 1601–1605. 10.1007/s12526-016-0591-1

[29] Araya JF, Aliaga JA, Opresko D. First record of *Alternatipathes bipinnata* (Cnidaria: Antipatharia) in the Southern Hemisphere. Zootaxa. 2017; 4312: 189–193. doi: 10.11646/zootaxa.4312.1.11

[30] Manso CLC, Prata J, Araya JF. Deep-water ophiuroids (Echinodermata) associated with anthozoans and hexactinellid sponges from northern Chile. Thalassas 2018; 34(1): 93–102. 10.1007/s41208-017-0042-1

[31] Araya JF, Bitner MA. Rediscovery of *Terebratulina austroamericana* Zezina, 1981 (Brachiopoda: Cancellothyrididae) from off northern Chile. Zootaxa. 2018; 4407: 443–446. doi: 10.11646/zootaxa.4407.3.11 29690189 10.11646/zootaxa.4407.3.11

[32] Folmer O, Black M, Hoeh W, Lutz R, Vrijenhoek R. DNA primers for amplification of mitochondrial cytochrome c oxidase subunit I from diverse metazoan invertebrates. Mol Mar Biol Biotechnol. 1994; 3: 294–299. 7881515

[33] Cowart DA, Huang C, Arnaud-Haond S, Carney SL, Fisher CR, Schaeffer SW. Restriction to large- scale gene flow versus regional panmixia among cold seep *Escarpia* spp. (Polychaeta, Siboglinidae). Mol Ecol. 2013; 22: 4147–4162. 10.1111/mec.12379 23879204

[34] Lane DJ. 16S/23S rRNA sequencing In: Stackebrandt E, Goodfellow M, editors. Nucleic acid techniques in bacterial systematics. New York: Wiley and Sons; 1991 pp. 115–175.

[35] Ronquist F, Huelsenbeck JP. MrBayes 3: Bayesian phylogenetic inference under mixed models. Bioinformatics. 2003; 19: 1572–1574. 10.1093/bioinformatics/btg180 12912839

[36] Lanfear R, Frandsen PB, Wright AM, Senfield T, Calcott B. PartitionFinder 2: new methods for selecting partitioned models of evolution for molecular and morphological phylogenetic analyses. Mol Biol Evol. 2016; 34: 772–773. 10.1093/molbev/msw260 28013191

[37] Stamatakis A. RAxML-VI-HPC: maximum likelihood-based phylogenetic analyses with thousands of taxa and mixed models. Bioinformatics. 2006; 22: 2688–2690. 10.1093/bioinformatics/btl446 16928733

[38] Katoh K, Standley DM. MAFFT multiple sequence alignment software version7: improvements in performance and usability. Mol Biol Evol. 2013; 30: 772–780. 10.1093/molbev/mst010 23329690 PMC3603318

[39] Thiel V, Hügler M, Blümel M, Baumann HI, Gärtner A, Schmaljohann R, Strauss H, Garbe-Schönberg, Petersen S, Cowart DA, Fisher CR, Imhoff JF. Widespread occurrence of two carbon fixation pathways in tubeworm endosymbionts: lessons from hydrothermal vent associated tubeworms from the Mediterranean Sea. Front Microbiol. 2012; 3: 423 10.3389/fmicb.2012.00423 23248622 PMC3522073

[40] McMullin ER, Hourdez S, Schaeffer SW, Fisher CR. Phylogeny and biogeography of deep sea vestimentiferan tubeworms and their bacterial symbionts. Symbiosis. 2003; 34: 1–41.

[41] Webb M. Studies on *Lamellibrachia barhami* (Pogonophora) II. The reproductive organs. Zool Jahrb Abt Anat Ontog Tiere. 1977; 97: 455–481.

[42] Southward EC, Tunnicliffe V, Black M. Revision of the species of *Ridgeia* from northeast Pacific hydrothermal vents, with a redescription of *Ridgeia piscesae* Jones (Pogonophora: Obturata = Vestimentifera). Can J Zool. 1995; 73: 282–295. 10.1139/z95-033

[43] Tunnicliffe V, St. Germain C, Hilário A. Phenotypic variation and fitness in a metapopulation of tubeworms (*Ridgeia piscesae* Jones) at hydrothermal vents. PLoS ONE 2014; 9: e110578 10.1371/journal.pone.0110578 25337895 PMC4206443

[44] Williams NA, Dixon DR, Southward EC, Holland PWH. Molecular evolution and diversification of the vestimentiferan tube worms. J Mar Biol Ass U.K. 1993; 73: 437–452. 10.1017/S0025315400032987

[45] Gail F, Hunt S. Tubes of deep-sea hydrothermal vent worms *Riftia pachyptila* (Vestimentifera) and *Alvinella pompejana* (Annelida). Mar Ecol Prog Ser. 1986; 3: 267–274.

[46] Shillito B, Lechaire J-P, Goffinet G, Gaill F. Composition and morphogenesis of the tubes of vestimentiferan worms. Geol Soc London Spec Publ. 1995; 87: 295–302. 10.1144/GSL.SP.1995.087.01.22

[47] Palmer ANS, Styan CA, Shearman DCA. Foot mucus is a good source for non-destructive genetic sampling in Polyplacophora. Conserv Genet. 2008; 9: 229–231. 10.1007/s10592-007-9320-4

[48] Ravaux J, Zbinden M, Voss-Foucart MF, Compère P, Goffinet G, Gail F. Comparative degradation rates of chitinous exoskeletons from deep-sea environments. Mar Biol. 2003; 143: 405–412. 10.1007/s00227-003-1086-8

[49] Hurtado LA, Lutz RA, Vrijenhoek RC. Distinct patterns of genetic differentiation among annelids of eastern Pacific hydrothermal vents. Mol Ecol. 2004; 13: 2603–2615. 10.1111/j.1365-294X.2004.02287.x 15315674

[50] Zhang H, Johnson SB, Flores VR, Vrijenhoek RC. Intergradation between discrete lineages of *Tevnia jerichonana*, a deep-sea hydrothermal vent tubeworm. Deep Sea Res Part II Top Stud Oceanogr. 2015; 121: 53–61. 10.1016/j.dsr2.2015.04.028

[51] Zardus JD, Etter RJ, Chase MR, Rex MA, Boyle EE. Bathymetric and geographic population structure in the pan-Atlantic deep-sea bivalve *Deminucula atacellana* (Schenck, 1939). Mol Ecol. 2006; 15: 639–651. 10.1111/j.1365-294X.2005.02832.x 16499691

[52] Georgieva MN, Wiklund H, Bell JB, Eilertsen MH, Mills RA, Little CTS, Glover AG. A chemosynthetic weed: the tubeworm *Sclerolinum contortum* is a bipolar, cosmopolitan species. BMC Evol Biol. 2015; 15: 280 10.1186/s12862-015-0559-y 26667806 PMC4678467

[53] Eilertsen MH, Georgieva MN, Kongsrud JA, Linse K, Wiklund H, Glover AG, Rapp HT.Genetic connectivity from the Arctic to the Antarctic: Sclerolinum contortum and Nicomache lokii (Annelida) are both widespread in reducing environments. Sci. Rep. 2018; 8: 4810 10.1038/s41598-018-23076-0 29556042 PMC5859262

[54] Kobayashi G, Mukai R, Alalykina I, Miura T, Kojima S. Phylogeography of benthic invertebrates in deep waters: a case study of *Sternaspis* cf. *williamsae* (Annelida: Sternaspidae) from the northwestern Pacific Ocean. Deep Sea Res Part II Top Stud Oceanogr. In press. 10.1016/j.dsr2.2017.12.016

[55] Govenar B, Le Bris N, Gollner S, Glanville J, Aperghis AB, Hourdez S, et al Epifaunal community structure associated with *Riftia pachyptila* aggregations in chemically different hydrothermal vent habitats. Mar Ecol Prog Ser. 2005; 305: 67–77. 10.3354/meps305067

[56] Govenar B, Fisher CR. Experimental evidence of habitat provision by aggregations of *Riftia pachyptila* at hydrothermal vents on the East Pacific Rise. Mar Ecol. 2007; 28: 3–14. 10.1111/j.1439-0485.2007.00148.x

[57] Maldonado M, Young CM. A new species of poecilosclerid sponge (Porifera) from bathyal methane seeps in the Gulf of Mexico. J Mar Biol Assoc U.K. 1998; 78: 795–806. 10.1017/S0025315400044787

[58] Miyake H, Hashimoto J, Chikuchishin M, Miura T. Scyphopolyps of *Sanderia malayensis* and *Aurelia aurita* attached to the tubes of vestimentiferan tubeworm (*Lamellibrachia satsuma*) at submarine fumaroles in Kagoshima Bay. Mar Biotechnol. 2004; 6: S174–8.

[59] Tunnicliffe V, Southward AJ. Growth and breeding of a primitive stalked barnacle *Leucolepas longa* (Cirripedia: Scalpellomorpha: Eolepadidae: Neolepadinae) inhabiting a volcanic seamount off Papua New Guinea. J Mar Biol Assoc UK. 2004; 84:121–32. 10.1017/S0025315404008987h

[60] Järnegren J, Tobias CR, Macko SA, Young CM. Egg predation fuels unique species association at deep-sea hydrocarbon seeps. Biol Bull. 2005; 209:87–93. 10.2307/3593126 16260768

[61] Desbruyères D, Segonzac M, Bright M. Handbook of deep-sea hydrothermal ventfauna 2nd ed Biologiezentrum: Linz; 2006.

[62] Sen Gupta BK, Smith LE, Lobegeier MK. Attachment of foraminifera to vestimentiferan tubeworms at cold seeps: refuge from seafloor hypoxia and sulfide toxicity. Ecol Monogr. 2007; 72: 365–82. 10.1016/j.marmicro.2006.06.007

[63] Becker EL, Cordes EE, Macko SA, Lee RW, Fisher CR. Using Stable isotope compositions of animal tissues to infer trophic interactions in Gulf of Mexico lower slope seep communities. PLoS ONE 2013; 8: e74459 10.1371/journal.pone.0074459 24324572 PMC3855623

[64] Kobayashi G, Kojima S. First record of *Protomystides hatsushimaensis* (Annelida: Phyllodocidae) inhabiting vacant tubes of vestimentiferan tubeworms. Mar Biodivers Rec. 2017;10: 25 10.1186/s41200-017-0127-9

[65] Forget NL, Perez M, Juniper SK. Molecular study of bacterial diversity within the trophosome of the vestimentiferan tubeworm *Ridgeia piscesae*. Mar Ecol. 2015; 36: 35–44. 10.1111/maec.12169

[66] Duperron S, De Beer D, Zbinden M, Boetius A, Schipani V, Kahil N, Gaill F. Molecular characterization of bacteria associated with the trophosome and the tube of *Lamellibrachia* sp., a siboglinid annelid from cold seeps in the eastern Mediterranean. FEMS Microbiol Ecol. 2009; 69: 395–409. 10.1111/j.1574-6941.2009.00724.x 19583785

[67] Sellanes J, Pedraza-García MJ, Zapata-Hernández G. ¿Las áreas de filtración de metano constituyen zonas de agregación del bacalao de profundidad (*Dissostichus eleginoides*) frente a Chile central? Lat Am J Aquat Res. 2012; 40: 980–991. 10.3856/vol40-issue4-fulltext-14

